# The Role of Hydrogen Peroxide in Mediating the Mechanical Wounding-Induced Freezing Tolerance in Wheat

**DOI:** 10.3389/fpls.2018.00327

**Published:** 2018-03-14

**Authors:** Tong Si, Xiao Wang, Chunzhao Zhao, Mei Huang, Jian Cai, Qin Zhou, Tingbo Dai, Dong Jiang

**Affiliations:** ^1^National Technique Innovation Center for Regional Wheat Production, Key Laboratory of Crop Physiology and Ecology in Southern China, Ministry of Agriculture, National Engineering and Technology Center for Information Agriculture, Nanjing Agricultural University, Nanjing, China; ^2^Department of Horticulture and Landscape Architecture, Purdue University, West Lafayette, IN, United States

**Keywords:** mechanical wounding, hydrogen peroxide, systemic wound response, wheat, freezing tolerance

## Abstract

Systemic wound response (SWR), a well-characterized systemic signaling response, plays crucial roles in plant defense responses. Progress in understanding of the SWR in abiotic stress has also been aided by the researchers. However, the function of SWR in freezing stress remains elusive. In this study, we showed that local mild mechanical wounding enhanced freezing tolerance in newly occurred systemic leaves of wheat plants (*Triticum aestivum* L.). Wounding significantly increased the maximal photochemical efficiency of photosystem II, net photosynthetic rate, and the activities of the antioxidant enzymes under freezing stress. Wounding also alleviated freezing-induced chlorophyll decomposition, electrolyte leakage, water lose, and membrane peroxidation. In addition, wounding-induced freezing stress mitigation was closely associated with the ratio between reduced glutathione (GSH) and oxidized glutathione (GSSG), and the ratio between ascorbate (AsA) and dehydroascorbate (DHA), as well as the contents of total soluble sugars and free amino acids. Importantly, pharmacological study showed that wounding-induced freezing tolerance was substantially arrested by pretreatment of wheat leaves with the scavenger of hydrogen peroxide (H_2_O_2_) or the inhibitor of NADPH oxidase (RBOH). These results support the hypothesis that local mechanical wounding-induced SWR in newly occurred leaves is largely attributed to RBOH-dependent H_2_O_2_ production, which may subsequently induce freezing tolerance in wheat plants. This mechanism may have a potential application to reduce the yield losses of wheat under late spring freezing conditions.

**Highlights**:

In our previous research, we found that local mechanical wounding could induce freezing tolerance in the upper systemic leaves of wheat plants. Surprisingly, in this paper, we further demonstrated that local mechanical wounding could also increase freezing resistance in newly occurred leaves of wheat plants. RBOH mediated H_2_O_2_ and ascorbate–glutathione cycle participate in this systemic wound response.

## Introduction

Plants are constantly exposed to various unfavorable environmental stresses in their natural habitat. Freezing stress (below 0°C) adversely affects the physiological and metabolic processes and significantly decreases grain yield and grain quality of temperate crop plants (Xin and Browse, 2000; Shinozaki et al., 2003; Puhakainen et al., 2004; Mittler, 2006). Winter wheat (*Triticum aestivum* L.), which originates from temperate regions, has been challenged by the frequent and untimely frost or the cold spell caused by the global climate change (IPCC, 2007). In the past decade, efforts have been made to understand the mechanism underlying the freezing tolerance in crops (Thomashow, 1999; Asseng et al., 2011; Rinalducci et al., 2011; Zhao et al., 2016). Cold acclimation is a complex process in plants that involves extensive physiological, biochemical, and metabolic changes (Thomashow, 1999; Chinnusamy et al., 2007; Zhao et al., 2017). Abiotic stress (e.g., UV-B radiation, chilling, and drought) priming and exogenous application of plant growth hormones (e.g., salicylic acid, jasmonic acid, and abscisic acid) have been widely adopted in cold acclimation to the acquisition of plant freezing tolerance (Yang et al., 2007; Kosová et al., 2012; Zhou et al., 2012; İşeri et al., 2013; Li et al., 2015b). However, more effective strategies to alleviate the freezing stress on crops are still unveiled.

Hydrogen peroxide (H_2_O_2_), as one of the main components of reactive oxygen species (ROS), is known to play a dual role in plant acclimation to freezing stress. Early researches have revealed the adverse effects of H_2_O_2_ in plants such as membrane lipid peroxidation, toxicity, and cell death under abiotic stresses. However, more recent studies have shed light on the role of H_2_O_2_ as a signaling molecule triggering the tolerant responses to abiotic stresses (Miller et al., 2009; Suzuki et al., 2012; Baxter et al., 2014). NADPH oxidase (respiratory burst oxidase homolog, RBOH) has been considered as a major source of H_2_O_2_ in these rapidly systemic signaling responses (Gilroy et al., 2014; Dietz et al., 2016). RBOH-mediated H_2_O_2_ production, known as an initial response to environmental stresses, is required for the translocation of the systemic signal to distant plant parts, thereby triggering responses of plants to cope with future stress events, including cold, heat, high light intensity, and salinity (Neill et al., 2002; Mittler et al., 2004; Sagi and Fluhr, 2006; Gaupels et al., 2016). Therefore, a deep investigation of the correlation between RBOH and the initiation of plant abiotic stress signal will be of far-reaching significance.

Wounding stresses lead to damage or loss of organs/tissues in plants (Nabity et al., 2009; Schmidt et al., 2015). Systemic wound response (SWR) is a systemic response caused by biotic (e.g., herbivore attack and pathogens infection) and abiotic (e.g., wind, hailing, heavy downpour, and the manmade touching such as mowing, trample, and stamping) stimuli. SWR has emerged as a very fruitful avenue for plants to tolerate multiple stresses (Sanchez-Serrano, 2001; Mousavi et al., 2013; Mittler and Blumwald, 2015; Guo et al., 2016). For example, wounding increases salt tolerance through the participation of Ca^2+^-dependent protein kinase in tomato (Capiati et al., 2006). SWR also contributes to the rapid synthesis of jasmonate to enhance plant resistance to herbivore and insect attacks (Schilmiller and Howe, 2005; Wasternack et al., 2006; Koo et al., 2009; Onkokesung et al., 2010). In winter wheat production, physical wounding has been widely applied to the seedlings at tillering stage for the acquisition of cold tolerance after jointing stage. However, the mechanisms underlying the interaction of physical wounding with low temperature remains obscure.

The potential role of SWR could be to alert the unstressed remote tissues to the upcoming stress events through systemic signaling and its function cannot be completed without the participation of H_2_O_2_ (Orozco-Cardenas and Ryan, 1999; León et al., 2001). Previous researches have well demonstrated the vital role of H_2_O_2_ in SWR in response to various stresses, but the knowledge about the role of SWR in freezing stress is scanty. In a field experiment, application of mechano-stimulation at critical developmental stages enhanced chilling tolerance in the newly occurred leaves of wheat (Li et al., 2015a), whereas its in-depth signaling transduction mechanism remains unclear. More recently, we further demonstrated that local mechanical wounding could induce freezing tolerance in upper systemic leaves, the process of which was mediated by H_2_O_2_ originated from RBOH (Si et al., 2017). Thus, the SWR-induced signaling and the RBOH-mediated H_2_O_2_ signaling seem to share the similar morphological and developmental patterns in plants. In wheat production, however, freezing stress usually occurs later in jointing stage after we take effective agronomic measures such as mechanical wounding. Our previous study revealed the short term alleviation effect of mechanical wounding on freezing stress, thereby it is natural to raise a question that whether RBOH-mediated H_2_O_2_ is a pivotal hub required for wounding-induced freezing tolerance in the newly occurred leaves (long term alleviation effect). Therefore, a new study was designed in purpose of exploring the alleviation effect of the local mechanical wounding on freezing stress in the newly occurred leaves. It was hypothesized that RBOH-mediated H_2_O_2_ could play a vital role in this SWR-induced freezing tolerance in wheat.

## Materials and Methods

### Plant Materials

Seeds of winter wheat (*Triticum aestivum* L. cv. Yangmai 16) were surface-sterilized with 2.5% sodium hypochlorite for 15 min, followed by five times rinse with sterile distilled water and then sown in vermiculites. When the second leaf fully expanded, the seedlings were transferred to the plastic containers (45 cm in length, 35 cm in width, and 18 cm in height) with half-strength Hoagland’s nutrient solution for hydroponic culture. The growth conditions were: photoperiod of 14/10 h (day/night) with photosynthetic photon flux density (PPFD) of around 600 μmol m^-2^s^-1^ during day time, relative air temperature of 25/18°C (day/night), relative humidity of ca. 80–90%. When the fifth leaves fully expanded, the seedlings were used for further treatments as described below.

### Experimental Design

One share of the wheat plants were pretreated with 5 mM dimethylthiourea (DMTU, a H_2_O_2_ and OH scavenger) or 100 μM diphenyleneiodonium (DPI, a RBOH inhibitor), while the rest share were treated with distilled water for 8 h. Half of the share of plants pretreated with water were remained under normal growth conditions as control, while the rest half of water pretreated plants and those pretreated with DMTU or DPI were wounded as described in our previous study (Si et al., 2017). In brief, the fifth leaves (termed local leaves) from the bottom of seedlings were wounded. The main vein in the center of the leaves was crushed five times using a hemostat and with interval of each injury of 1.0 cm. Wounding did not cause any visible alterations. All plants were kept in normal growth conditions after wounding. Around 10 days after wounding, the sixth leaves (termed systemic leaves) fully expanded. All of the wounded plants and half of the unwounded plants were exposed to cold treatment in the climate chamber for 24 h at 4/-2°C (day/night) with otherwise unchanged growing conditions. After cold treatment, the temperature was set back to 25/18°C (day/night) for 24-h recovery. Measurements were performed at the end of recovery period on the systemic leaves. Both the local and the systemic leaves were harvested at the same time for biochemical analysis. Twenty-four plants were used for each treatment with three replicates each.

### Leaf Gas Exchange, Chlorophyll Fluorescence Parameters, and Total Chlorophyll Content

Gas exchange parameters were performed on the systemic leaves with a portable photosynthesis system (LI-6400, LI-COR Inc., United States). The photosynthetically active radiation (PAR), ambient CO_2_ concentration, air temperature, and relative humidity used in the measurement were 800 μmol m^-2^ s^-1^, 380 μmol mol^-1^, 25°C and 80%, respectively. The plants were recovered at 25°C for 1 h after freezing treatment before measurements of gas exchange parameters. Measurements of chlorophyll fluorescence parameters were taken using the same leaves for gas exchange with a chlorophyll fluorescence imaging system (CF Imager; Technologica Ltd., United Kingdom) as described previously (Li et al., 2014). The whole plants were dark-adapted in the same growth conditions for 30 min before imaging. All treatments were imaged with the same leaf position as area of interest and analyzed using the FluorImager software (Version 2.2; Technologica Ltd., United Kingdom). For total chlorophyll content, 0.1 g fresh leaf was extracted in 50 mL of pigment extraction solution containing anhydrous ethanol and acetone (1:1, v/v) at 25°C in the dark for 12 h. Then the supernatant was measured for absorbance at 647 and 663 nm. Content of total chlorophyll was calculated according to Lichtenthaler and Wellburn (1983).

### Content of H_2_O_2_, Membrane Injury, and Lipid Peroxidation

H_2_O_2_ production was monitored by the histochemical staining method according to Thordal-Christensen et al. (1997) with some modifications. In brief, leaf samples were immediately immersed in 1 mg mL^-1^ 3,3 -diaminobenzidine (DAB) solution (dissolved in 50 mM Tris-acetate, pH 3.8), vacuum-infiltrated for 10 min and incubated for 12 h at 25°C in dark. Then, the leaves were bleached in 95% (v/v) boiled alcohol for approximately 15 min until the brown spots were visualized. Photographs were taken with a stereo microscope system (IX71, Olympus Co., Tokyo, Japan). The other branch of the samples were taken immediately in the liquid nitrogen to fix the concentration of H_2_O_2_. The quantification of H_2_O_2_ was assayed by monitoring the absorbance of titanium peroxide complex at 410 nm as reported previously (Willekens et al., 1997; Moloi and van der Westhuizen, 2006).

Membrane injury was estimated by relative electrolytic conductivity (REC) and relative water content (RWC) of leaf. REC was conducted according to Griffith and Mclntyre (1993) with some modifications. The conductivity was measured before (C1) and after (C2) the leaves were boiled in 10 mL distilled water for 30 min using a conductivity bridge (DDS-307A, LEX Instruments Co., Ltd., China). REC was then calculated as the division of C1 to C2 multiplied by 100 (REC (%) = C1/C2 × 100). RWC was measured due to the method of Jensen et al. (2000) and Rampino et al. (2006) with minor modifications. The whole leaves were excised and fresh weight (FW) was immediately recorded. Then the leaves were soaked for 4 h in distilled water at room temperature to record the turgid weight (TW). Total dry weight (DW) was recorded after drying in the oven for 24 h at 85°C. RWC was calculated based on the equation as RWC (%) = [(FW - DW)/(TW - DW)] × 100.

Lipid peroxidation was determined based on the thiobarbituric acid (TCA) reaction by measuring the amount of malondialdehyde (MDA) as described by Hodges et al. (1999). MDA content was calculated at 532 nm by subtracting the absorbance at 600 nm.

### Activities of the Antioxidant Enzymes

The extract for determination of antioxidant enzymes was prepared according to the method of Knörzer et al. (1996) with some improvements. Frozen leaf tissues (0.5 g FW) were ground using ice-cold mortar and pestle with 5 mL 25 mM HEPES-NaOH buffer (pH 7.8). The components of the buffer were as follows: 20% (v/v) glycerol, 1 mM ascorbic acid (AsA), 1 mM ethylenediaminetetraacetic acid (EDTA), 5 mM MgCl_2_, 1 mM dithiothreitol (DTT), and 1 mM reducing glutathione (GSH). The homogenates were centrifuged at 4°C and 12,000 × *g* for 20 min and the resulting supernatants were collected for enzyme analysis. The protein content were determined prior to the measurement of antioxidant enzymes according to Bradford (1976). Superoxide dismutase (SOD) activity was determined based on measurement of the reduction of nitro-blue tetrazolium at 560 nm using the method of Stewart and Bewley (1980). Ascorbate peroxidase (APX) activity was assayed by a decline at 290 nm as originally described by Nakano and Asada (1981) with some modifications. Catalase (CAT) activity was modified as a decrease at 240 nm as described by Patra et al. (1978). Glutathione reductase (GR) activity was assayed depending on the rate of decrease in the absorbance of NADPH at 340 nm as mentioned by Foyer and Halliwell (1976).

### Cell Walls Extraction and Activities of Cell-Wall Peroxidase

Cell walls were prepared according to the method of Lee and Lin (1995) by homogenizing the leaves (0.5 g) in ice-cold 50 mM phosphate buffer (pH 5.8). The homogenate was centrifuged at 1,000 × *g* and 4°C for 10 min and washed with the same buffer for three times. The pellet was collected for the determination of cell-wall peroxidase (POD) activities. POD activities were measured using a modification of the procedure as originally described by Ishida et al. (1987). The assay mixture was as follows: 50 μM NADH prepared in 30 mM Na-acetate buffer (pH 6.5), 5 mM MnCl_2_ and 20 μM p-coumaric acid. The reaction was started by adding the pellet, and then the decrease of absorbance at 340 nm by oxidizing NADH was measured.

### Contents of Ascorbate and Glutathione

Leaf tissue (0.2 g) was homogenized in 1.5 mL of ice-cold 5% metaphosphoric acid. The homogenate was centrifuged at 12,000 × *g* and 4°C for 15 min. The supernatant was used for analysis of ascorbate (AsA) according to previous research (Zhang and Kirkham, 1996) with appropriate modifications. Contents of total AsA and AsA were determined by a spectrophotometer at 265 nm using a standard curve. Dehydroascorbate (DHA) was calculated by subtracting AsA from the total AsA.

Fluorometric analysis was taken to estimate the reduced GSH and oxidized glutathione (GSSG) according to previous methods (Rao and Ormrod, 1995; Aravind and Prasad, 2005). Briefly, leaf tissue (0.2 g) was ground in 200 μL of 25% metaphosphoric acid containing 2 mM phosphate-EDTA (pH 8.0). After centrifugation at 12,000 × *g* and 4°C for 15 min, the supernatant was used for the measurement of GSH and GSSG at excitation 350 nm and emission 420 nm.

### Contents of Total Soluble Sugars and Free Amino Acids

One hundred milligrams of oven-dried leaf sample was powdered, and then extracted twice with 80% (v/v) ethanol at 80°C for 10 min and centrifuged at 3,000 × *g* for 30 min. The supernatant was then collected for measurements of contents of soluble sugars and free amino acids. The total soluble sugars content was assayed at 620 nm using the anthrone method according to the protocol described previously (Buysse and Merckx, 1993). Content of free amino acids was determined by the ninhydrin reaction at 570 nm improved by the method of Moore and Stein (1954).

### Total RNA Extraction and Quantitative Real-Time PCR (qRT-PCR)

Total RNA was extracted from wheat leaves using the Trizol reagent (Sangon, Shanghai, China), according to the manufacturer’s procedure. Total RNA (500 ng) was reverse transcribed to first-strand cDNA using a kit (Takara, Shiga, Japan). The gene-specific qRT-PCR primers were designed based on their cDNA sequences (Supplementary Table S1). The qRT-PCR analysis was performed using the SYBR Green PCR Master Mix (Takara, Shiga, Japan) on an iCycleri Q^TM^ real-time PCR detection system (Bio-Rad, Hercules, CA, United States). Each reaction (20.0 μL) consisted of 10.0 μL of SYBR Green PCR Master Mix, 2.0 μL of diluted cDNA and 0.1 μM forward and reserve primers. The PCR cycling conditions were as follows: 95°C for 5 min, followed by 39 cycles of denaturation at 95°C for 10 s and annealing at 60°C for 30 s. The wheat *Actin* gene was used as an internal control. The quantification of mRNA levels was calculated according to Livak and Schmittgen (2001).

### Statistical Analysis

All data were subjected to one-way analysis of variance (ANOVA) and tested for significant differences between treatments by the SPSS software (version 11.0; SPSS Inc., Chicago, IL, United States). The treatment effects were evaluated by a Tukey’s test (*P* < 0.05).

## Results

### Effects of Mechanical Wounding on Relative Electrolytic Conductivity and Relative Water Content Under Freezing

**FIGURE 1:**
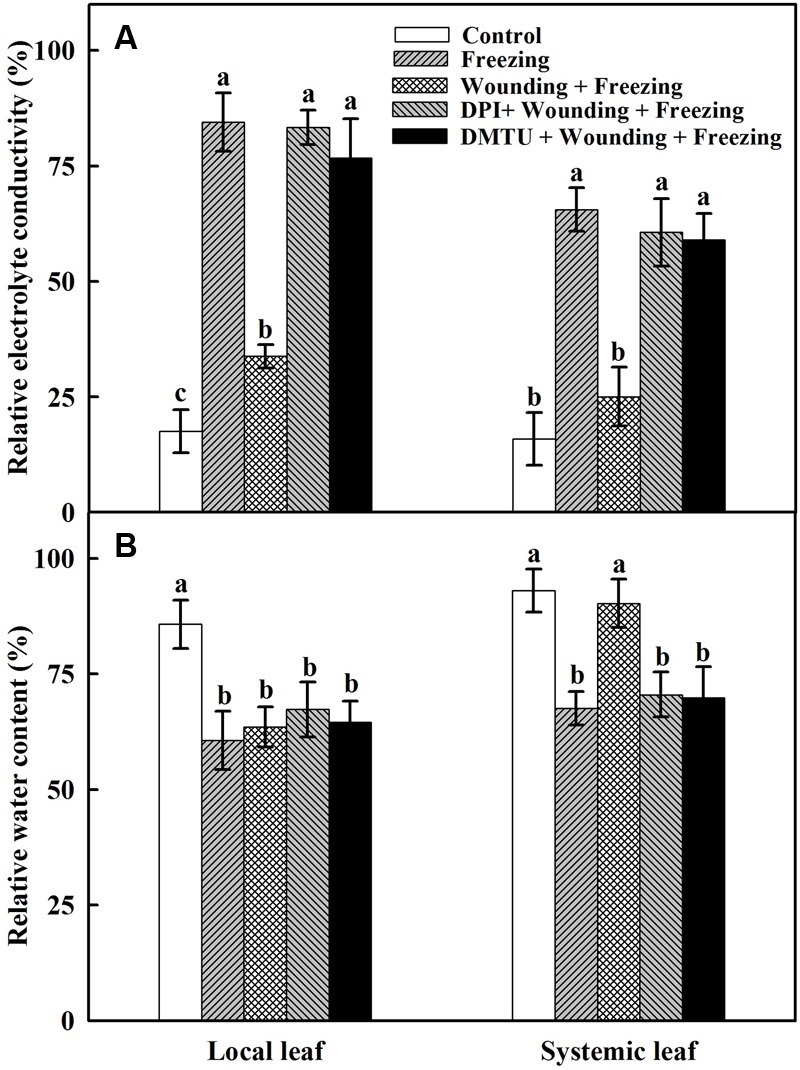
Effects of mechanical wounding on relative electrolytic conductivity (REC) and relative water content (RWC) in the local and the systemic leaves of wheat under freezing stress. REC **(A)** and RWC **(B)**. The local (fifth) leaves were separately pre-treated with distilled water, 5 mM DMTU or 100 μM DPI prior to wounding. At 10 days after wounding, the seedlings were exposed to freezing stress for 24 h. After 24 h recovery, REC and RWC were analyzed immediately on both local and systemic (sixth) leaves. Data are mean values ± SD of three independent replicates. Means denoted by different letters indicated significant difference at *P* < 0.05 according to Tukey’s test.

To assess the potential role of local mechanical wounding on freezing tolerance in the newly occurred systemic leaves and to understand the contribution of H_2_O_2_ in this process, wheat seedlings were exposed to freezing stress at 4/-2°C (day/night) for 24 h after being wounded and sprayed with inhibitor or scavenger of H_2_O_2_. The degree of leaf damage caused by freezing stress was assessed according to the changes of REC and RWC. Plants treated with freezing alone showed higher REC (**Figure 1A**) and lower RWC (**Figure 1B**) in both local and systemic leaves compared with the control seedlings. In contrast, REC was lower but RWC was higher in plants treated with wounding followed by freezing treatment in systemic leaves. In addition, the REC was significantly increased while RWC was decreased in plants pretreated with DMTU or DPI prior to wounding compared with control plants in response to freezing stress (**Figure 1**). These results confirmed that local mechanical wounding could reduce leaf damage in newly occurred systemic leaves caused by freezing stress, the process of which, to some extent, relies on the participation of H_2_O_2_.

### Effects of Mechanical Wounding on Gas Exchange Under Freezing

**FIGURE 2:**
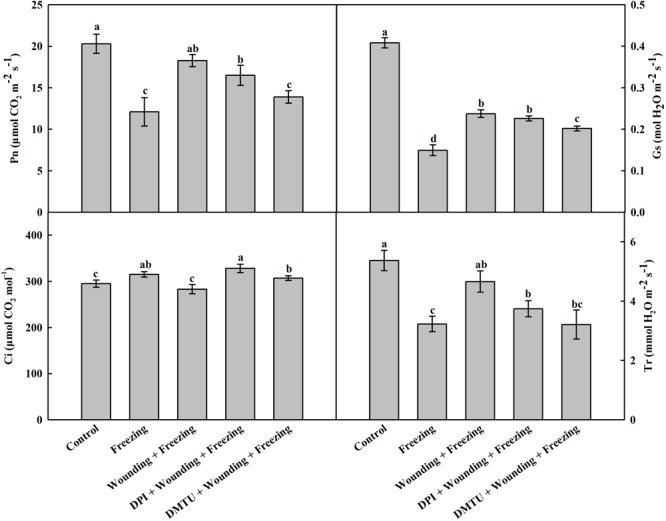
Effect of mechanical wounding on gas exchange in the local and the systemic leaves of wheat under freezing stress. Pn, photosynthetic rate; Gs, leaf stomatal conductance; Ci, internal CO_2_ content; Tr, transpiration rate. The local (fifth) leaves were separately pre-treated with distilled water, 5 mM DMTU or 100 μM DPI prior to wounding. At 10 days after wounding, the seedlings were exposed to freezing stress for 24 h. After 24 h recovery, gas exchange parameters were measured on the systemic leaves with a portable photosynthesis system. Data are mean values ± SD of three independent replicates. Means denoted by different letters indicated significant difference at *P* < 0.05 according to Tukey’s test.

Freezing stress has a negative effect on gas exchange of wheat leaves. Likewise, freezing significantly decreased the net photosynthetic rate (Pn), leaf stomatal conductance (Gs), and transpiration rate (Tr), but slightly increased internal CO_2_ concentration (Ci). Compared with the plants treated with freezing alone, the plants pretreated with wounding prior to being subjected to freezing showed increased levels of Pn, Gs, and Tr while decreased level of Ci in the systemic leaves (**Figure 2**). Notably, pretreatment of plants with DMTU or DPI prior to wounding effectively blocked wounding-induced Pn, Gs, and Tr, but elevated Ci under freezing stress. The results indicate that H_2_O_2_ is involved in wounding-induced alleviation effects on photosynthetic system in response to cold stress.

### Effects of Mechanical Wounding on Chlorophyll Fluorescence and Total Chlorophyll Contents Under Freezing

**FIGURE 3:**
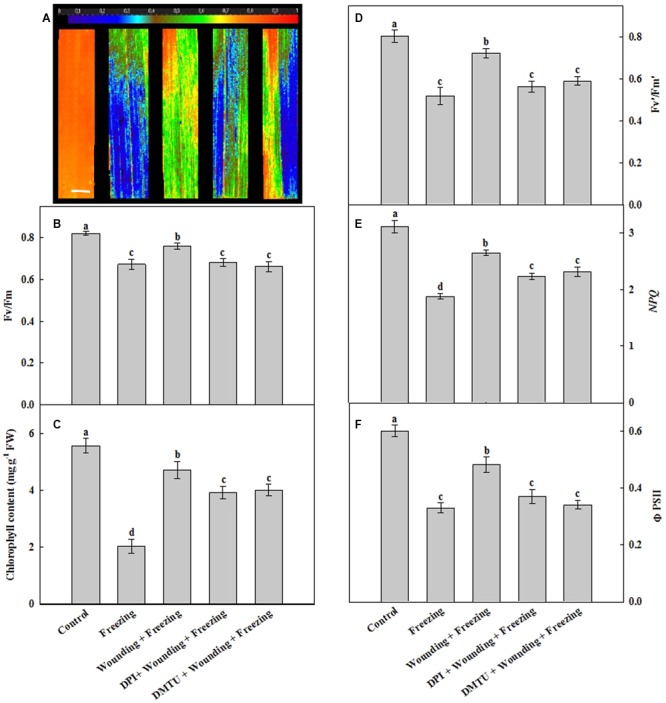
Effects of mechanical wounding on chlorophyll fluorescence parameters and chlorophyll content in the local and the systemic leaves of wheat under freezing stress. The maximal photochemical efficiency of photosystem II (PSII) as indicated by Fv/Fm **(A,B)**, total chlorophyll content expressed in mg g^-1^ FW (fresh weight) **(C)**, photochemical activity of PSII as indicated by Fv′/Fm′**(D)**, the non-photochemical quenching as indicated by *NPQ* **(E)** and the quantum yield of PSII as indicated by ΦPSII **(F)**. The local (fifth) leaves were separately pre-treated with distilled water, 5 mM DMTU or 100 μM DPI prior to wounding. At 10 days after wounding, the seedlings were exposed to freezing stress for 24 h. Chlorophyll fluorescence parameters were observed on the systemic (sixth) leaf as area of interest after 24 h recovery. Data are mean values ± SD of three independent replicates. Means denoted by different letters indicated significant difference at *P* < 0.05 according to Tukey’s test. Bar, 10 mm.

Freezing significantly decreased the maximal photochemical efficiency of photosystem II (Fv/Fm) of the newly occurred leaves (systemic leaves). In contrast, the plants subjected to wounding in local leaves prior to freezing treatment showed increased Fv/Fm in the systemic leaves under freezing as compared with the non-wounding treatment. For the seedlings pretreated with DMTU or DPI prior to wounding, Fv/Fm was as low as that of freezing stress treatment alone when compared with the control seedlings (**Figures 3A,B**). Freezing led to a decrease in total chlorophyll content while the plants pretreated with wounding restored the total chlorophyll content of systemic leaves. Treatment of leaves with DMTU or DPI prior to wounding also contributed to the maintenance of the chlorophyll content in response to freezing but the chlorophyll content was still slightly lower than that of wounding alone treatment under freezing (**Figure 3C**).

The PSII photochemical activity (indicated by Fv′/Fm′), the non-photochemical quenching (*NPQ*) and the quantum yield of PSII photochemistry (indicated by ΦPSII) in seedlings treated with freezing only and pretreated with DMTU or DPI prior to wounding were significantly lower than those in the control plants (**Figures 3D–F**). In contrast, Fv′/Fm′, *NPQ* and ΦPSII were still maintained at high levels in seedlings treated with wounding before low temperature treatment. Again, the leaves pretreated with DMTU or DPI prior to wounding attenuated the accelerating effects of wounding increased Fv′/Fm′, *NPQ* and ΦPSII under freezing stress. These results suggest that the H_2_O_2_ originated from local mechanical wounding plays an essential role in the maintenance of the function of PSII, reversely dispersing H_2_O_2_ with its scavengers eliminates this function under freezing stress.

### Effects of Mechanical Wounding on Membranes Lipid Peroxidation and H_2_O_2_ Content Under Freezing

**FIGURE 4:**
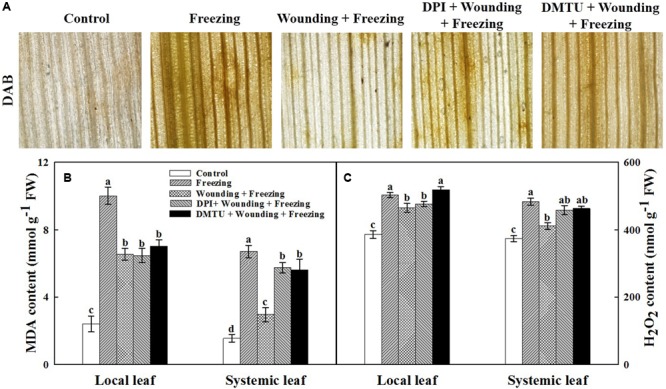
Effects of mechanical wounding on the accumulation of H_2_O_2_ and content of malondialdehyde in the local and the systemic leaves of wheat under freezing stress. DAB staining **(A)**, Malondialdehyde (MDA) content expressed in mmol g^-1^ FW (fresh weight) **(B)** and H_2_O_2_ content expressed in nmol g^-1^ FW **(C)**. The local (fifth) leaves were separately pre-treated with distilled water, 5 mM DMTU or 100 μM DPI prior to wounding. At 10 days after wounding, the seedlings were exposed to freezing stress for 24 h. After 24 h recovery, 3,3-diaminobenzidine (DAB) staining was used to detect the presence of H_2_O_2_ in systemic leaves. Dark brown spots represent localized H_2_O_2_ accumulation. The leaves were also collected immediately to determine the contents of H_2_O_2_ and MDA. Data are mean values ± SD of three independent replicates. Means denoted by different letters indicated significant difference at *P* < 0.05 according to Tukey’s test.

The oxidative damage and membranes lipid peroxidation in terms of contents of H_2_O_2_ and MDA were analyzed. Consistent with the damage observed in PSII, plants treated with freezing alone showed higher H_2_O_2_ content (**Figures 4A,C**) and MDA content (**Figure 4B**) in both local and systemic leaves compared with the control seedlings. In contrast, H_2_O_2_ content and MDA content were lower in plants treated with wounding followed by freezing treatment in systemic leaves. It was noticeable that plants pretreated with DMTU or DPI prior to wounding had higher levels of H_2_O_2_ and MDA contents in both local and systemic leaves compared with control plants in response to freezing (**Figure 4**). These results reveal a novel role of mechanical wounding in alleviating oxidative damage and membranes lipid peroxidation on systemic leaves under freezing stress.

### Source of H_2_O_2_ in Response to Wounding and Freezing

**FIGURE 5:**
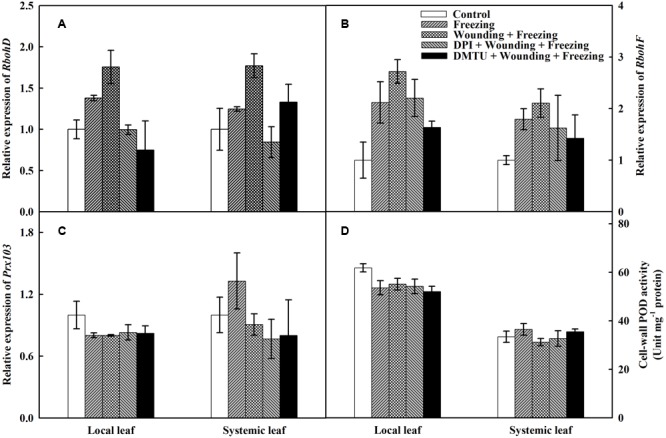
Effects of mechanical wounding on relative transcript abundance of *Rbohs, Prx103* and activities of cell-wall POD in the local and the systemic leaves of wheat under freezing stress. Relative transcript abundance of *RbohD* **(A)**, *RbohF* **(B)**, *Prx103* **(C)**, and activities of cell-wall POD **(D)**. The local (fifth) leaves were separately pre-treated with distilled water, 5 mM DMTU or 100 μM DPI prior to wounding. At 10 days after wounding, the seedlings were exposed to freezing stress for 24 h. After 24 h recovery, both local and systemic (sixth) leaves were harvested for the measurement.

To further characterize the possible source of H_2_O_2_ under wounding and freezing, the expression of two partially redundant NADPH oxidase catalytic subunit genes, *RbohD* and *RbohF* were analyzed. The expression of *RbohD* was up-regulated by both freezing and wounding treatments in local leaves compared with control, whereas an up-regulation of the *RbohD* was only observed in “wounding + freezing” treatment in systemic leaves (**Figure 5A**). Wounding and freezing treatments both significantly up-regulated the expression of *RbohF* in local and systemic leaves (**Figure 5B**). In contrast, no difference was found on either *RbohD* or *RbohF* expression in DPI or DMTU pretreatment plants in local and systemic leaves compared with control.

Cell-wall POD is associated with plant abiotic stresses and is responsible for the accumulation of H_2_O_2_. The expression of *Prx103*, representative of the PODs, was significantly depressed in “freezing” and “wounding + freezing” treatments of local leaves, whereas no significant difference was observed in that of systemic leaves. Conversely, exogenous DPI or DMTU pretreatments had no impact on the expression of *Prx103* in either local or systemic leaves (**Figure 5C**). The cell-wall POD activity was significantly decreased under wounding and freezing treatments in local leaves. Pretreatment of DPI or DMTU also had a decline of POD activity in local leaves under freezing stress. However, no difference between each treatments was observed in systemic leaves (**Figure 5D**). The data so far indicate that plasma membrane NADPH oxidase but not cell-wall POD is the major source of H_2_O_2_ in wounding and freezing response.

### Effects of Mechanical Wounding on the Activities of Antioxidative Enzymes Under Freezing

**FIGURE 6:**
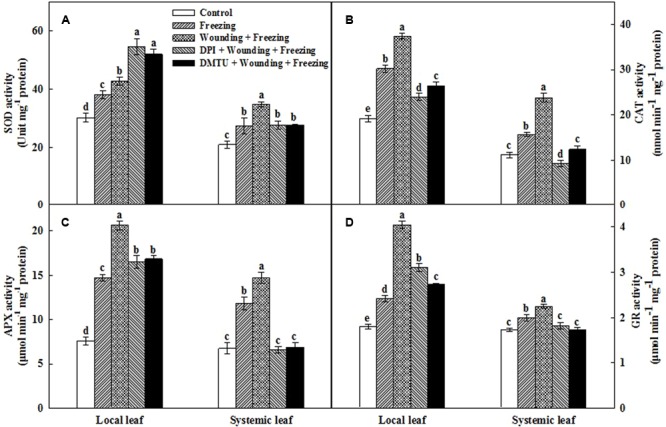
Effect of mechanical wounding on activities of the antioxidant enzymes in the local and the systemic leaves of wheat under freezing stress. Activities of superoxide dismutase (SOD) **(A)**, catalase (CAT) **(B)**, ascorbate peroxidase (APX) **(C)**, and glutathione reductase (GR) **(D)**. The local (fifth) leaves were separately pre-treated with distilled water, 5 mM DMTU or 100 μM DPI prior to wounding. At 10 days after wounding, the seedlings were exposed to freezing stress for 24 h. After 24 h recovery, both local and systemic (sixth) leaves were collected for the measurement. Data are mean values ± SD for three independent replicates. Means denoted by different letters indicated significant difference at *P* < 0.05 according to Tukey’s test.

The antioxidant defense machinery is of paramount importance in protecting plants against oxidative damages. Analysis of four key antioxidant enzymes showed that the activities of SOD, CAT, APX, and GR were significantly increased in local and systemic leaves after 24 h cold treatment (**Figure 6**). In particular, the induction of the activities of all these four antioxidant enzymes were more pronounced in the plants pretreated with wounding than the plants treated with freezing alone. Pretreatment of plants with DMTU or DPI prior to wounding, to a large extent, reduced the activities of these enzymes in both local and systemic leaves under freezing stress. One exception is that DMTU and DPI significantly increased the activity of SOD in local leaves compared with other treatments (**Figure 6A**). Analysis of relative gene expression showed that the *Mn SOD, Cu/Zn SOD, CAT, APX*, and *GR* transcription levels displayed similar patterns to the changes in their respective enzyme activities with a few exceptions (Supplementary Figure S1). Collectively, we demonstrated that local mechanical wounding enhanced antioxidant systems in response to freezing stress, to some extent, with the participation of H_2_O_2_.

### Effects of Mechanical Wounding on Ascorbate–Glutathione Cycle Under Freezing

**FIGURE 7:**
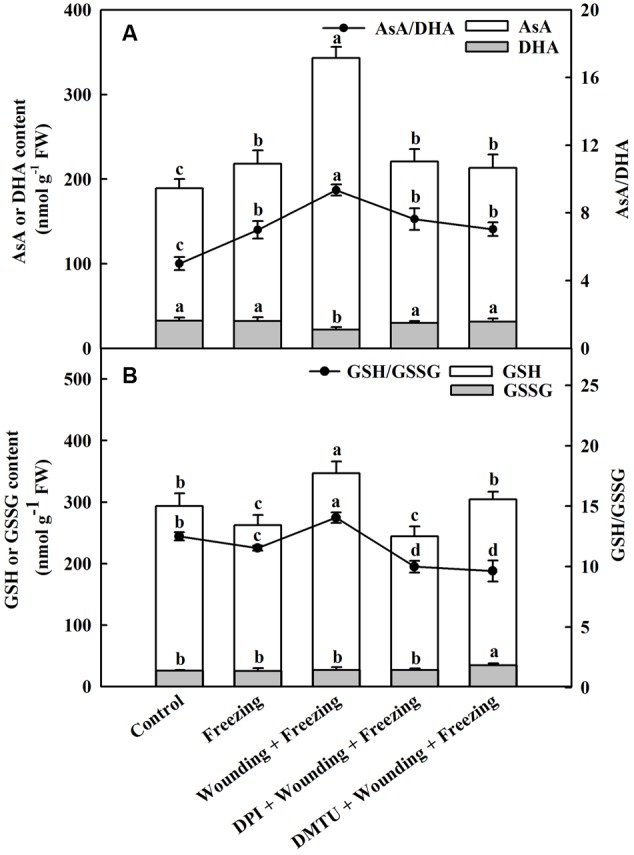
Effect of mechanical wounding on AsA–GSH cycle in the systemic leaves of wheat under freezing stress. Ascorbate (AsA) and dehydroascorbate (DHA) content, ratio of AsA to DHA **(A)**. Glutathione (GSH) and glutathione disulfide (GSSG) content, ratio of GSH to GSSG **(B)**. The local (fifth) leaves were separately pre-treated with distilled water, 5 mM DMTU or 100 μM DPI prior to wounding. At 10 days after wounding, the seedlings were exposed to freezing stress for 24 h. After 24 h recovery, the systemic (sixth) leaves were collected and AsA, DHA, GSH, and GSSG contents were determined. Data are mean values ± SD for three independent replicates. Means denoted by different letters indicated significant difference at *P* < 0.05 according to Tukey’s test.

Exposing to freezing conditions dramatically elevated the content of AsA in the systemic leaves of wheat plants. The decrease of DHA content was observed in plants treated with wounding alone compared to other treatments under freezing. Similarly, the wounding-induced production of AsA was blocked by DPI or DMTU application. The ratio between AsA and DHA was significantly increased in the plants pretreated with wounding while pretreatment of plants with DMTU or DPI prior to wounding had little effects on AsA/DHA compared to control (**Figure 7A**). The content of GSH was decreased in plants only treated with freezing, but elevated in plants pretreated with wounding. The glutathione disulfide (GSSG) content was not significantly changed in response to wounding and freezing except for DMTU-pretreated plants. A similar response was observed for the ratio between GSH and GSSG. Freezing combined with pre-wounding treatment led to increased ratio of GSH/GSSG, whereas plants pretreated with DPI or DMTU showed lower ratio of GSH/GSSG compared with the plants treated with freezing alone (**Figure 7B**). Analysis of transcription levels of key enzymes in the AsA–GSH cycle showed that freezing combined with pre-wounding treatment significantly up-regulated *MDAR* in both local and systemic leaves while up-regulated *DHAR* only in systemic leaves compared to other treatments. Freezing treatment alone also increased the expression of *MDAR* and *DHAR* in systemic leaves but the increment was not statistically significant. In plants pretreated with DPI or DMTU, however, no significant differences were found between these treatments and control in the expression of *MDAR* and *DHAR* (Supplementary Figure S2).

### Effects of Mechanical Wounding on Contents of Total Soluble Sugars and Free Amino Acids Under Freezing

**FIGURE 8:**
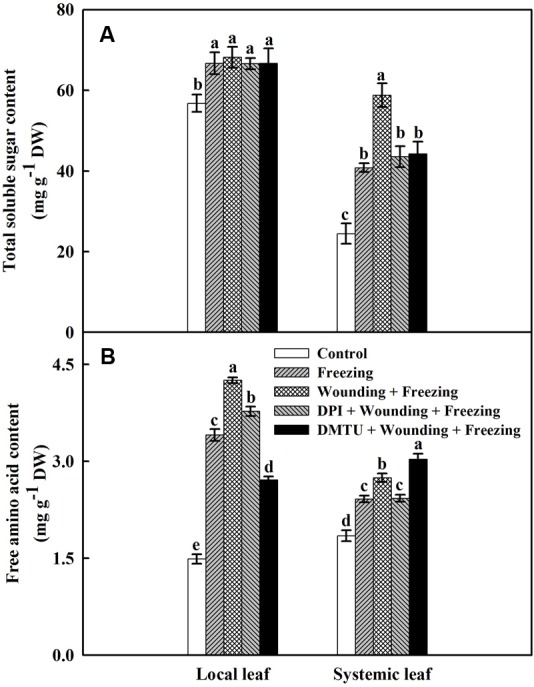
Effects of mechanical wounding on contents of total soluble sugars and free amino acids in the local and the systemic leaves of wheat under freezing stress. Total soluble sugars content expressed in mg g^-1^ DW (dry weight) **(A)** and free amino acids content expressed in mg g^-1^ DW **(B)**. The local (fifth) leaves were separately pre-treated with distilled water, 5 mM DMTU or 100 μM DPI prior to wounding. At 10 days after wounding, the seedlings were exposed to freezing stress for 24 h. After 24 h recovery, both local and systemic (sixth) leaves were collected and contents of total soluble sugars and free amino acids were determined. Data are mean values ± SD for three independent replicates. Means denoted by different letters indicated significant difference at *P* < 0.05 according to Tukey’s test.

Both wounding and freezing treatments induced accumulations of soluble sugars and free amino acids in local and systemic leaves of wheat plants (**Figure 8**). For the total soluble sugars content, no significant differences were found between the plants treated with DPI or DMTU and the plants treated with wounding alone under freezing. However, freezing combined with pre-wounding treatment had higher content of total soluble sugars in the systemic leaves than the plants treated with freezing alone. The wounding-induced increase of total soluble sugars content was restored in plants pretreated with DPI or DMTU (**Figure 8A**). Similarly, the plants treated with wounding alone also had a higher level of free amino acids compared to other treatments in both local and systemic leaves except in plants pretreated with DMTU (**Figure 8B**). Thus, the elevation of two effective osmolytes, total soluble sugars and free amino acids, is essential for wounding-mediated acquisition of freezing tolerance in wheat plants.

## Discussion

To date, studies on the positive effects of SWR mainly focused on biotic stresses, with scant attention to abiotic stresses. Here, we present evidence showing that local mechanical wounding enhances freezing tolerance in newly occurred systemic leaves of wheat plants. Wounding enhanced gas exchange and photochemistry performances, reduced membranes lipid peroxidation, increased activities of antioxidant enzymes, probably by raising the ratio of AsA/DHA and GSH/GSSG, and elevating the level of osmotic adjustment substance. The RBOH-mediated production of H_2_O_2_ plays a vital role in this SWR as evidenced by the results that application of DPI or DMTU which inhibits the production of H_2_O_2_, at least partially, blocked wounding-induced freezing tolerance.

Chlorophyll fluorescence and the contents of photosynthetic pigments have been widely considered as good indicators for the assessment of stress tolerance in plants (Fracheboud et al., 1999; Kalaji et al., 2016). In our study, freezing inhibited the activity of PSII as indicated by the decrease of Fv/Fm, Fv′/Fm′, *NPQ*, and ΦPSII (**Figure 3**). Moreover, local mechanical wounding alleviated the inhibition of freezing on PSII, which suggests that wounding treatment might directly affect the activity of PSII. Surprisingly, the application of the inhibitor or scavenger of H_2_O_2_ in local leaves before wounding had offset the protective effects of wounding on PSII in systemic leaves, thus providing clues for us to dissect the relationship among wounding, H_2_O_2_, and freezing. Previous studies have indicated that H_2_O_2_ is one of the universal signals responding to wounding (Minibayeva et al., 2009; Soares et al., 2011). H_2_O_2_ also acts as a systemic signal to alert systemic tissues to respond and adapt to the upcoming cold stress (Miller et al., 2009; Si et al., 2017). Our quantitative data showed that H_2_O_2_ bursts after freezing stress. Surprisingly, wounding treatment significantly reduced the accumulation of H_2_O_2_ in the systemic leaves compared to freezing control (**Figures 4A,C**). Furthermore, pretreatment of plants with DPI or DMTU had similar effects on the inhibition of chlorophyll fluorescence, hinting a possibility that RBOH is the major source of H_2_O_2_. Intensive studies have revealed the diverse functions of *RbohD* and *RbohF* at the heart of plant signaling processes and defense responses (Kwak et al., 2003; Chaouch et al., 2012; Marino et al., 2012). In **Figure 5**, we showed that *RbohD* and *RbohF* were highly expressed in both local and systemic leaves after wounding and freezing stress. In contrast, no significant differences on the cell-wall POD activities and the transcription of *Prx103* were observed. Therefore, the accumulation of H_2_O_2_ could be originated from RBOH in this SWR.

Previous studies also revealed that pretreatment of plants with appropriate content of H_2_O_2_ enhanced cold stress in different plant species (Yu et al., 2003; Mora-Herrera et al., 2005; İşeri et al., 2013). At first glance, H_2_O_2_ plays a direct role in wounding-induced protection of PSII. Our study, nevertheless, showed that the inhibition of PSII caused by freezing was not attenuated by the pretreatment of plants with exogenous H_2_O_2_ (data not shown). The contradiction between the previous and our results may due to the different temperatures used in the experiments. Previous papers used chilling temperature (>0°C) as cold treatment, but we used freezing temperature (<0°C). One possible explanation is that the combination of exogenous H_2_O_2_ and freezing may cause multiple stresses to wheat seedlings. In accordance with the chlorophyll fluorescence data, the chlorophyll contents showed the same trend with those parameters of chlorophyll fluorescence (**Figure 3C**). These observations imply that wounding-induced H_2_O_2_, mainly originated from RBOH, functions as a signal molecule that indirectly triggers the protection of PSII under freezing stress.

Net photosynthetic rate (Pn) is another indicator of plant abiotic stress (Rossel et al., 2007). In our study, the Pn value was highly decreased after freezing stress, whereas local mechanical wounding partially restored Pn in systemic leaves compared with freezing control. Again, pretreatment of local leaves with DPI or DMTU diminished the positive effect of wounding on Pn (**Figure 2**). It is worth mentioning that the decrease of Pn occurred concomitantly with the decreases of the parameters of chlorophyll fluorescence, such as Fv/Fm, Fv′/Fm′, *NPQ*, and ΦPSII. Therefore, we speculated that the reduction in photosynthesis was mainly attributable to the photoinhibition during freezing process. Similar results have been stated by earlier reports, which showed that photoinhibition is the major limitation of photosynthesis under cold stress (Krivosheeva et al., 1996; Allen and Ort, 2001). In brief, it is highly likely that local mechanical wounding strengthens the ability of the plants to maximize their photosynthetic performance by reducing the photo-oxidation in systemic leaves.

The inhibition of photosynthesis could be attributed to stomatal and/or non-stomatal limitations during multiple abiotic stresses (Jones, 1985; Flexas and Medrano, 2002; Yu et al., 2009; Varone et al., 2012). In our study, Ci showed a reverse tendency of change compared to Gs in all treatments (**Figure 2**), suggesting that non-stomatal limitations occurred under freezing stress. The Pn, Gs, and Tr were reduced, but the Ci was increased after the application of DPI or DMTU, hinting a possibility that H_2_O_2_ is involved in wounding induced photoinhibition. Freezing responses are associated with the changes in antioxidant system (Gulen et al., 2009; Zhou et al., 2012). Consistent with earlier studies, we found that the activities of antioxidant enzymes, such as SOD, CAT, APX, and GR, were increased after wounding and freezing treatment. The activities of these four enzymes were highly correlated with the changes of photosystem parameters in systemic leaves (**Figure 6**). Furthermore, the activities of these four enzymes were blocked by the application of DPI or DMTU. Hence, H_2_O_2_ plays a profound role in the alleviation of photoinhibition and membrane peroxidation under freezing stress induced by wounding.

Cellular GSH redox state is well-known to regulate a variety of biological processes and the activities of proteins through thiol group modification (Neill et al., 2002; Moreno et al., 2008; Jiang et al., 2012). Plants can change the GSH redox status to cope with the changing environment. To investigate the mechanism underlying the ameliorating effect of mechanical wounding on freezing induced photosystem inhibition and oxidative stress, the AsA–GSH cycle was analyzed, which showed that wounding led to increases in both AsA and GSH levels in systemic leaves, and ultimately increases the ratio of AsA/DHA. GSH is an electron donor that is essential for the conversion of DHA to AsA (Noctor et al., 2012; Sun et al., 2015). We provided strong evidence that H_2_O_2_ plays an important role in maintaining GSH pools induced by wounding, which finally leads to higher ratio of GSH/GSSG. Pretreatment of plants with DPI or DMTU significantly decreased wounding-induced contents of AsA and GSH in response to freezing stress. This result is consistent with the antioxidant enzymes analysis data, illustrating that H_2_O_2_ seems to be a regulatory molecule that affects the AsA–GSH cycle in wounding-induced freezing tolerance. GR is not only an antioxidant enzyme but also responsible for reproducing GSH from GSSG (Buchanan and Balmer, 2005). When the plants treated with DPI or DMTU before wounding, GR activity and the ratio of GSH/GSSG exhibited a sharp decrease in the systemic leaves after freezing stress. Totally, we deduce that the initially elevated H_2_O_2_ by local mechanical wounding could act as a systemic signal to trigger the reduction of the ratio of GSSG/GSH in systemic leaves, the process of which is probably mediated by enhanced activity of GR. Besides, wounding up-regulated *MDAR* and *DHAR* may also participate in maintaining GSH pools under freezing stress, which was consistant with the increased ratio of AsA/DHA and GSH/GSSG (**Figure 7** and Supplementary Figure S2). Previous studies have indicated that cellular GSH redox state plays an important role in the synthesis of Calvin cycle enzymes, which ultimately influence the photosystem (Irihimovitch and Shapira, 2000; Dietz, 2008). Consistent with previous studies, we observed that the levels of AsA/DHA and GSH/GSSG in plants treated with wounding and freezing were significantly higher than those in control plants (**Figure 7**). This is consistent with the photosynthesis data where local mechanical wounding helps to maintain the Pn level under freezing stress (**Figure 2**). In total, modulation of the cellular GSH redox state is probably required for the control of the photosynthesis in wounding induced freezing tolerance.

The potential roles of soluble sugars and free amino acids in the cold response have been widely studied in plants (Less and Galili, 2008; Tarkowski and Van Den Ende, 2015). As a major component of soluble sugars, sucrose can protect cell membranes by directly interacting with the phosphate in their lipid headgroups, and thus decreases membrane permeability under freezing stress (Strauss and Hauser, 1986; Furuya et al., 2014). Consistent with these findings, we observed a sharp increase of total soluble sugars in both local and systemic leaves after wounding and/or freezing stress (**Figure 8A**). The increment was more significant in freezing combined with pre-wounding treatment than the plants with other treatments, including pretreatment with DPI or DMTU. These results indicate that H_2_O_2_-mediated local mechanical wounding is involved in wounding-induced total soluble sugars under freezing stress. During photosynthesis, more than 80% of the assimilated CO_2_ contributes to the synthesis of sucrose (Koch, 2004). Coincidentally, photosynthesis significantly increased after wounding under freezing stress in systemic leaves (**Figure 2**). Thus, the accumulation of total soluble sugars could be interpreted as the result of enhanced photosynthesis. Furthermore, carbohydrates like sucrose have generally higher scavenging ability against ROS in synergism than other components (Couée et al., 2006; Tarkowski and Van Den Ende, 2015). The activities of antioxidant enzymes as well as the levels of AsA/DHA and GSH/GSSG in plants treated with wounding and freezing were maintained at high levels compared with freezing control, which may help to increase the threshold level of plant freezing tolerance together with total soluble sugars. As another effective osmolyte, free amino acids also take part in several metabolic processes such as chlorophyll synthesis, cell osmotic pressure regulation, and maintenance of protein stability (Rai, 2002; Kovács et al., 2011). The similar changes of free amino acids were observed as that of total soluble sugars (**Figure 8B**). Therefore, wounding-induced free amino acids may act synergistically with total soluble sugars in combating freezing stress.

## Conclusion

**FIGURE 9:**
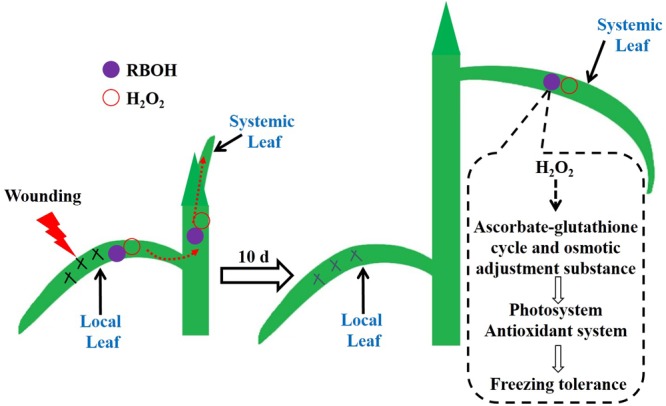
The working model of the mechanical wounding on the local leaves enhancing tolerance to freezing of the newly occurred systemic leaves in wheat. Wounding on the local leaves triggers the accumulation of H_2_O_2_ via the NADPH oxidase (RBOH) pathway. The burst of H_2_O_2_ functions as a crucial signal molecule being further conducted and amplified in the systemic leaves, which consequently initiates the anti-freezing responses in terms of stimulated antioxidant capacity, accumulation of osmolytes and improved photosynthesis in the systemic leaves.

We found that local mechanical wounding elevated the H_2_O_2_ level in systemic leaves mediated by RBOH. H_2_O_2_ may drive the AsA/DHA and GSH/GSSG cycles toward a more reduced state, and meanwhile accumulate osmolytes such as soluble sugars and free amino acids, which in turn enhance the photosystem and antioxidant system to mitigate oxidative states in the newly occurred leaves during freezing stress (**Figure 9**). Additional experiments are needed to provide proteomic and genetic evidences of the involvement of RBOH in wounding-induced freezing tolerance. Furthermore, determination of the interactions of H_2_O_2_ with several vital plant hormones such as abscisic acid, jasmonic acid, and salicylic acid would be of great interest to elucidate this long-distance signal transduction cascade.

## Author Contributions

TS and DJ conceived and designed the experiments. TS, XW, CZ, MH, JC, QZ, and TD performed the experiments. TS and DJ wrote the article with contribution of all the authors.

## Conflict of Interest Statement

The authors declare that the research was conducted in the absence of any commercial or financial relationships that could be construed as a potential conflict of interest.
