# Supplementary material for: The Role of Hydrogen Peroxide in Mediating the Mechanical Wounding-Induced Freezing Tolerance in Wheat
Source: Front Plant Sci. 2018 Mar 14;9:327. doi: 10.3389/fpls.2018.00327 (PMC5861560; doi:10.3389/fpls.2018.00327)
Supplement: Supplementary file 1 [file Data_Sheet_1.docx]

Supplementary Material

**Title:** The Role of Hydrogen Peroxide in Mediating the Mechanical Wounding-Induced Freezing Tolerance in Wheat

**Authors:** Tong Si^1,2^, Xiao Wang^1^, Chunzhao Zhao^2^, Mei Huang^1^, Jian Cai^1*^, Qin Zhou^1^, Tingbo Dai^1^, Dong Jiang^1 **^

**^*^Co-correspondence author:** Jian Cai: caijian@njau.edu.cn

**^**^Correspondence author:** Dong Jiang: jiangd@njau.edu.cn


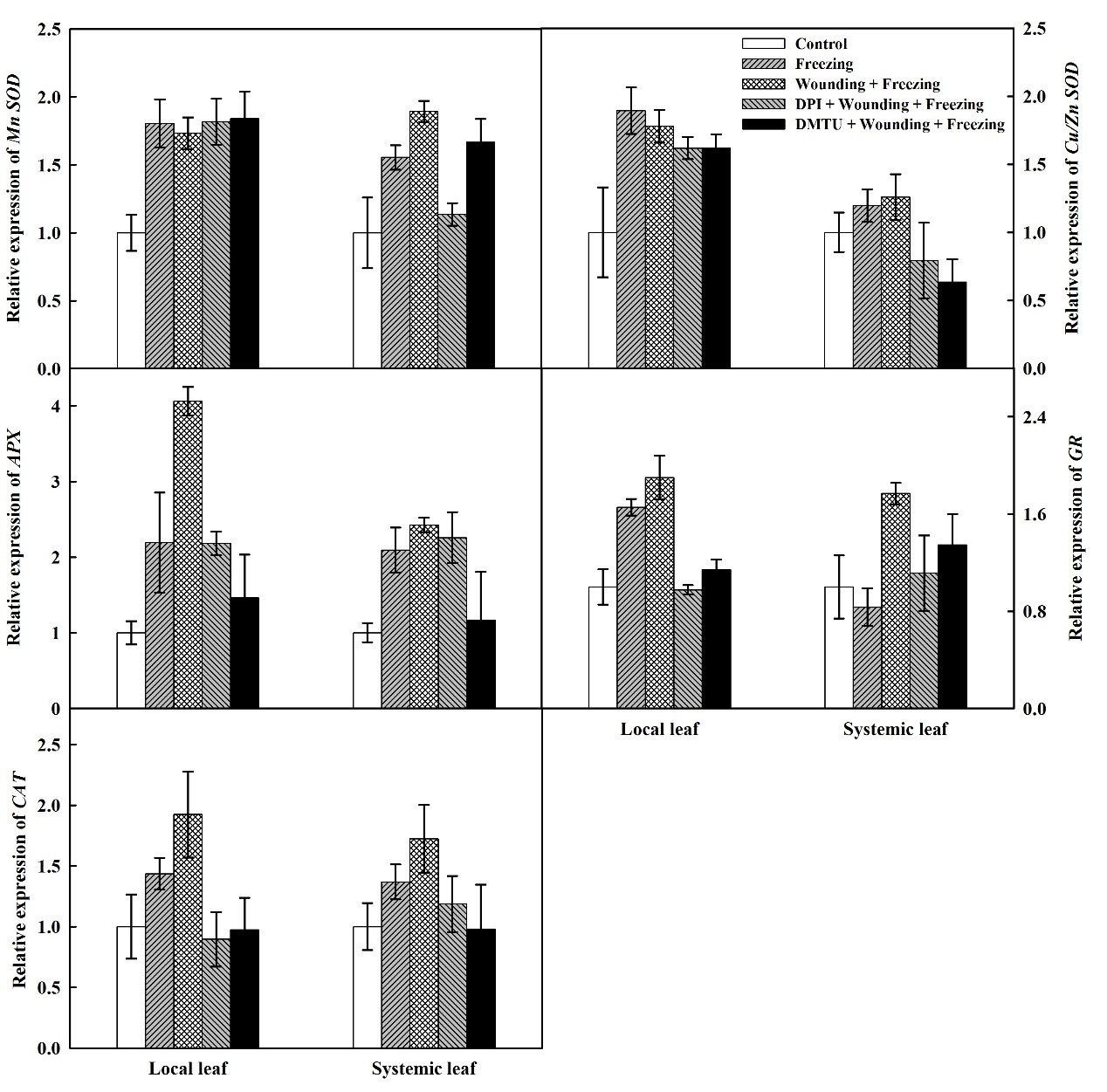


**Supplementary Figure S1** Effect of mechanical wounding on relative transcript abundance of *Mn SOD*, *Cu/Zn SOD*, *APX*, *GR* and *CAT* in the local and the systemic leaves of wheat under freezing stress. The local (fifth) leaves were separately pre-treated with distilled water, 5 mM DMTU or 100 µM DPI prior to wounding. At 10 d after wounding, the seedlings were exposed to freezing stress for 24 h. After 24 h recovery, both local and systemic (sixth) leaves were harvested for the analysis of the relative expression of antioxidant genes.


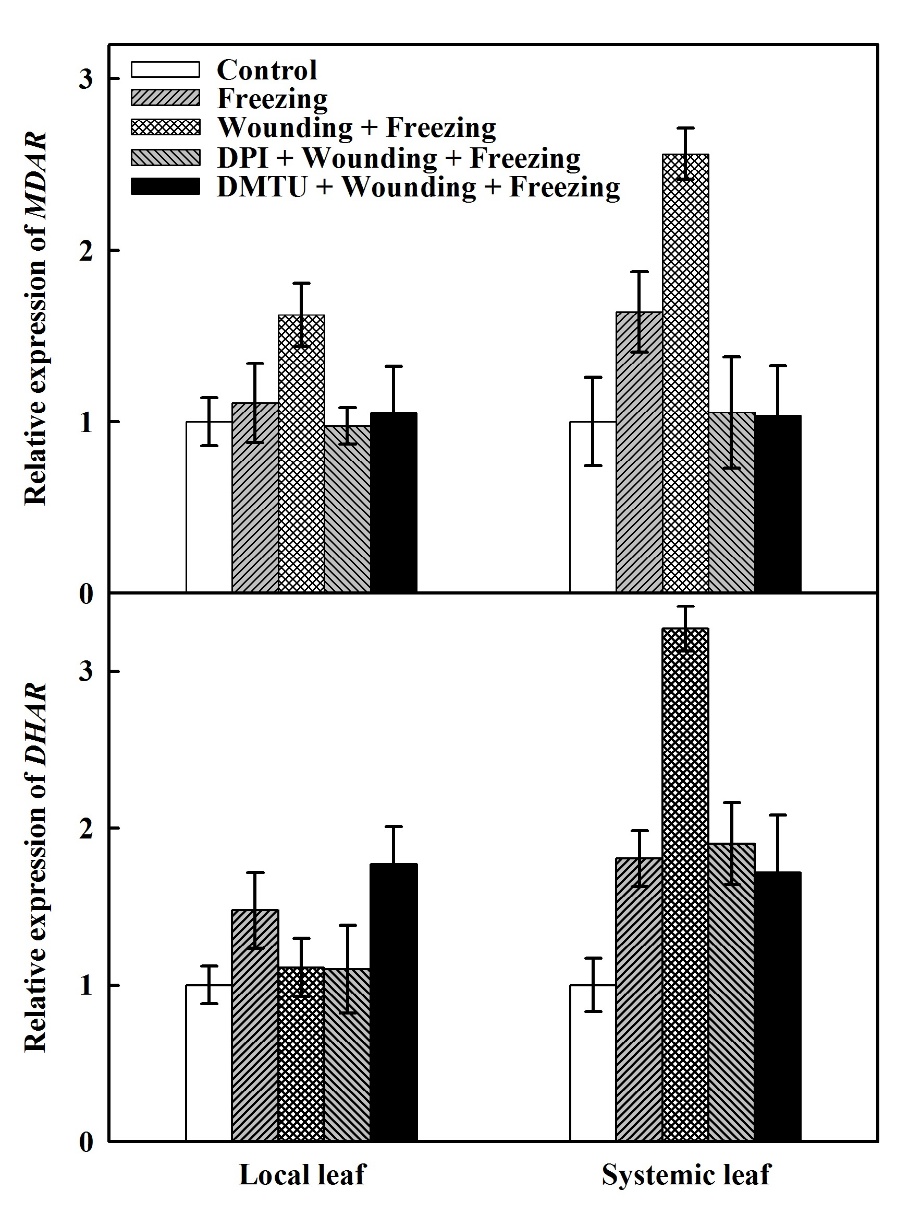


**Supplementary Figure S2** Effect of mechanical wounding on relative transcript abundance of *MDAR* and *DHAR* in the local and the systemic leaves of wheat under freezing stress. The local (fifth) leaves were separately pre-treated with distilled water, 5 mM DMTU or 100 µM DPI prior to wounding. At 10 d after wounding, the seedlings were exposed to freezing stress for 24 h. After 24 h recovery, both local and systemic (sixth) leaves were harvested for the analysis of the relative expression of antioxidant genes.

**Supplementary Table S1** Gene-specific primers designed for qRT-PCR.

| Gene | Forward sequence (5’-3’) | Reverse sequence (5’-3’) | Amplicon size (bp) |
| --- | --- | --- | --- |
| *Actin* | GCTCTCCAACAACATTGCCAAC | GCTTCTGCCTGTCACATACGC | 165 |
| *Mn SOD* | CCGGACTACCTGACCAACATC | CCAACAGCGGGAAACTCAA | 135 |
| *Cu/Zn SOD* | TGGGAGAGCGTTTGTTGTTC | GTCTTCCACCAGCATTTCCA | 92 |
| *CAT* | CCATGAGATCAAGGCCATCT | ATCTTACATGCTCGGCTTGG | 103 |
| *APX* | AAAACCACCTACTGCCACCCTATC | AGCATTCGCTCCATGACTCAACT | 148 |
| *GR* | TGCGTCCCGAAGAAGATACT | GTTGATGTCCCCGTTGATCT | 96 |
| *RbohD* | ACCACCAGACCAGACCAGAC | TGGTTGGATAGGAGGCGTAG | 70 |
| *RbohF* | TGGCACCCCTTCTCAATTAC | CTCTCGTGTCCAGTCACCAA | 84 |
| *Prx103* | GCATACTAGCCAGCACGACA | ACACGGTTTCAAGAGCTGGT | 85 |
| *MDAR* | AGAAGTTTACGCCCTTCGGC | TTGGAATGTCATCGCCATC | 132 |
| *DHAR* | GTGCCTGTGTATAACGGTG | ACAAGTGATGGAGTTGGGT | 94 |
